# Wafer‐Scale Atomically‐Thin Gold: Transferable Platform for Flexible Optoelectronics, Thermal Management and Bio‐Interfaces

**DOI:** 10.1002/advs.76495

**Published:** 2026-08-04

**Authors:** Dmitry Yakubovsky, Mikhail Mironov, Georgy Ermolaev, Aleksandr Khrebtov, Aleksandr Slavich, Prashant Narute, Pritom Chowdhury, Roman Kirtaev, Igor Khramtsov, Anton Minnekhanov, Gleb Tselikov, Dmitriy Grudinin, Maria Sidorova, Nikolay Pak, Pavel Maslov, Andrey Vyshnevyy, Hongkai Zhou, Vasyl G. Kravets, Alexander N. Grigorenko, Ivan Kruglov, Nikita Orekhov, Dmitry Kireev, Aleksey Arsenin, Kostya S. Novoselov, Valentyn Volkov

**Affiliations:** ^1^ Emerging Technologies Research Center XPANCEO Dubai United Arab Emirates; ^2^ Department of Biomedical Engineering University of Massachusetts Amherst Amherst MA USA; ^3^ Department of Physics and Astronomy University of Manchester Manchester UK; ^4^ The University of Manchester National Graphene Institute Manchester UK; ^5^ Department of Materials Science and Engineering National University of Singapore Singapore Singapore; ^6^ Institute for Functional Intelligent Materials National University of Singapore Singapore Singapore

**Keywords:** epidermal sensors, flexible optoelectronics, thermal camouflage, transparent electrodes, ultrathin gold films

## Abstract

The integration of metals into two‐dimensional material architectures is essential for next‐generation electronics, but their high surface energy intrinsically favors three‐dimensional island growth, hindering the scalable fabrication of continuous, atomically‐thin metallic layers. While recent advances have yielded freestanding 2D metals like goldene, their technological integration remains constrained by limited dimensions. Here we resolve this fundamental thermodynamic challenge by merging template stripping with a graphene‐inspired transfer method to produce 6‐inch wafer‐scale, continuous, and transferable gold films with thicknesses approaching the atomic limit. These ultrathin gold films exhibit atomic smoothness with a root‐mean‐square roughness below 0.4 nm and possess near‐bulk electronic properties, yielding exceptional optoelectronic performance with optical transmittance above 86% and sheet resistance below 20 Ω/□. The adhesion‐free nature of the films enables their versatile integration into various devices, which we demonstrate with flexible organic light‐emitting diodes, effective thermal camouflage, and conformal epidermal sensors that achieve a signal‐to‐noise ratio of ≈15 dB for electrocardiogram and ≈70 dB for electromyogram monitoring, superior to that of clinical gel electrodes. The suggested approach establishes a universal and scalable route for integrating atomically‐thin metals into complex heterostructures and functional systems.

## Graphical abstract

Combining template stripping with a graphene‐inspired transfer yields continuous, transferable gold films at 6‐inch wafer scale with thicknesses approaching the atomic limit. The atomically smooth, near‐bulk‐quality films offer above 86% transmittance and sub‐20 Ω/square sheet resistance, serving as a universal platform for flexible optoelectronics, thermal management, and bio‐interfaces.

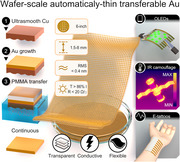

## 1 Introduction

The transformative impact of graphene and other two‐dimensional (2D) van der Waals materials has redefined the landscape of material sciences and condensed matter physics [1]. A frontier in this revolution is the extension of atomic‐scale fabrication principles to non‐van der Waals materials, particularly metals, to create a new class of quasi‐2D materials [2, 3, 4]. The ability to produce large‐area, transferable, and atomically thin metallic layers promises to unlock the next‐generation technologies, from transparent and flexible optoelectronics [4, 5] to thermal management [6, 7] and bio‐interfaces [8, 9]. However, realization of this vision had been prevented by a fundamental thermodynamic laws of ultrathin metals synthesis process [10, 11]. The primary problem is an extremely high surface energy of metals reaching ≈1120 mJ/m^2^ for gold [12], which is an order of magnitude larger than that of conventional 2D materials (compare, for instance with a typical value of ≈48 mJ/m^2^ for graphene under ambient conditions [13]). As a result, the standard growth mechanism of ultrathin gold follows the three‐dimensional (3D) island formation (the Volmer‐Weber growth mode) instead of layer‐by‐layer formation (the Frank‐van der Merwe growth mode), required for realization of ultrathin gold [14]. This thermodynamic challenge bifurcated the research into two distinct but ultimately constrained paths. The first approach focuses on engineering metal‐substrate interactions, using seed layers or adhesion promoters to enforce 2D layer‐by‐layer growth mechanism and suppress 3D island formation [11, 15, 16, 17, 18, 19]. While this method yielded high‐quality, continuous films, it usually comes at the cost of permanently bonding the metal to its growth substrate, thereby sacrificing the transferability essential for device integration. Even when transferability is achieved, as in prior work [4] where polymer‐assisted processes inspired by 2D materials science was used, the ultimate quality of the films is often compromised by the high roughness inherited from the underlying growth substrate [4]. In the present work, we remove this limitation by replacing the evaporated copper layer, whose topography is governed by its own grain structure, with an atomically smooth template‐stripped copper foil, and by replacing geometry‐constrained electrochemical delamination with the direct isotropic dissolution of a freestanding foil. These two changes lower the minimum continuous thickness from 3.5 to 1.5 nm, increase the peak transmittance from 80 to 86.6%, and scale the process from centimetre‐scale samples to full 6‐inch wafers. The second approach pursues the direct synthesis of 2D metals, such as monolayer goldene exfoliated from MAX‐phase precursors [3, 20] or other metals confined via van der Waals squeezing [2]. These milestone achievements indeed have produced true monolayer metals, but the resulting materials are limited to micrometer‐scale flakes or require encapsulation or stabilizing environments, precluding their use as large‐area, open‐surface components in wafer‐scale technologies. Consequently, a general strategy to produce continuous, transferable, and technologically relevant ultrathin metals has remained elusive.

Here, we resolve this challenge by creating a hybrid fabrication paradigm that synergistically merges techniques from two different fields: template stripping [21, 22] and polymer‐assisted transfer from 2D materials science [4]. Initially, our approach leverages upon template stripping to fabricate an atomically smooth wafer‐scale copper foil. Such pristine metallic surface allows us to overcome the thermodynamic barrier for the 2D growth of ultrathin gold, guiding the physical vapour deposition of gold into continuous metallic films. We then employ a robust, graphene‐inspired transfer‐process to cleanly delaminate this ultrathin gold film, rendering it a freestanding, handable layer that can be integrated with arbitrary substrates. The suggested two‐step process results in a film formation that unites the previously mutually exclusive goals of atomic‐scale thickness and large‐area transferability. We validate the versatility of this approach by fabricating adhesion‐free, wafer‐scale, and atomically smooth ultrathin gold films with high electrical conductivity of less than 20 Ω/□ and optical transparency above 86%, and seamlessly integrating them into high‐performance representative systems, including organic light‐emitting diodes (OLEDs), dynamic infrared thermal camouflage devices, and flexible epidermal electronics.

## 2 Results

### 2.1 Engineering Metal‐Substrate Energetics for Layer‐by‐Layer Gold Deposition

**FIGURE 1:**
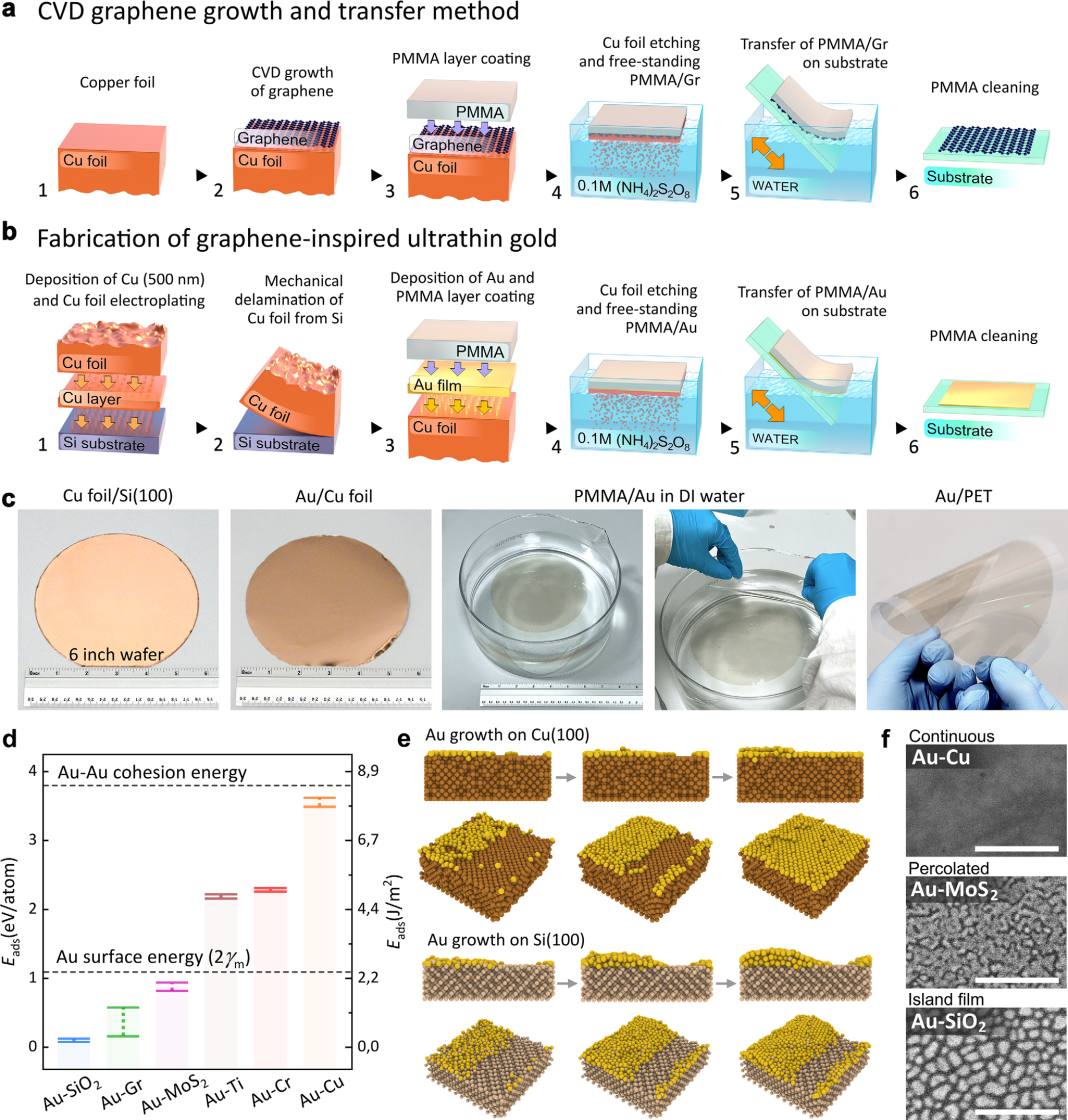
Ultrathin gold fabrication technology. Schematic illustration comparing a, the standard CVD graphene growth and transfer procedure with b, the graphene‐inspired method, which includes the preparation of a wafer‐scale ultrasmooth copper foil via a template‐stripping technique, followed by the growth and transfer of an ultrathin gold film. c, Photographs demonstrating sequential stages of graphene‐inspired transfer of a 5 nm‐thick ultrathin gold film onto a flexible polyethylene terephthalate (PET) substrate at the 6 inch‐scale wafer. d, Diagram of gold adatom adsorption energies to different materials, in comparison with the surface free energy and cohesive energy of gold (taken from literature data) [12, 23, 24, 25, 27, 28, 29, 30, 31, 32, 33]. e, Molecular dynamics simulation of anisotropic Au deposition: Au atoms are deposited only on the left half of the surface, demonstrating different growth behavior of Au layers on Cu vs. Si substrates. f, HR‐SEM surface images of 3 nm‐thick gold films grown on different substrates (scale bars: 100 nm).

**FIGURE 2:**
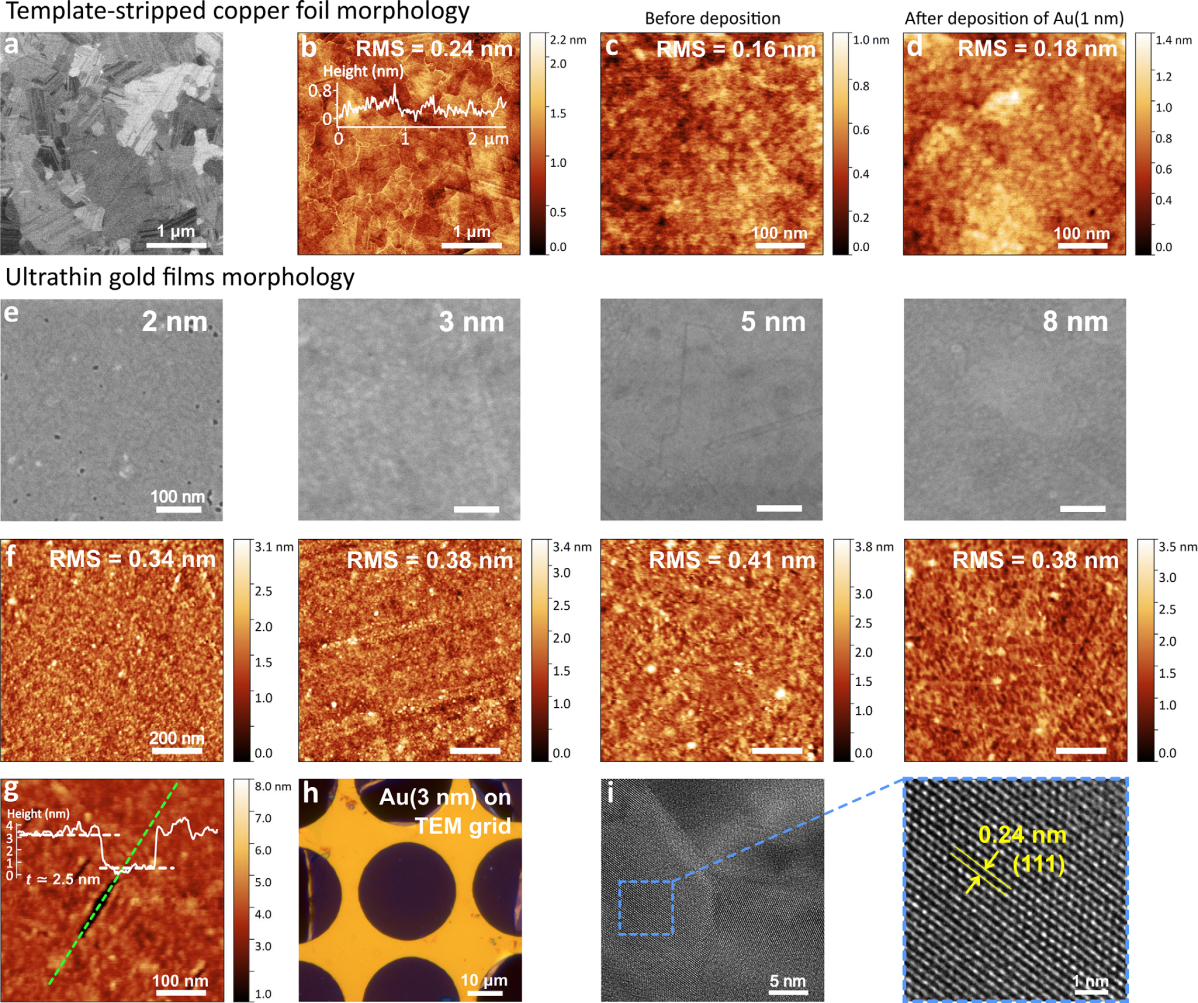
Structural and morphological characterization of the copper substrate and ultrathin gold films. a, HR‐SEM image of a copper foil prepared by template stripping. b, AFM topography map of the copper surface (4 × 4 µm^2^), with a height profile and RMS roughness value shown in the inset. AFM scans at a smaller scale (0.5 × 0.5 µm^2^) demonstrating the smoothness of c, the bare copper foil and d, the copper foil after 1 nm‐thick gold deposition. e, HR‐SEM images and f, AFM scans of ultrathin gold films with thicknesses ranging from 2 to 8 nm, transferred onto a SiO_2_/Si substrate, with RMS roughness values determined over 1 × 1 µm^2^ areas. g, AFM thickness measurement of 2 nm‐thick gold film on SiO_2_/Si, with a height profile taken from the cracked region. h, Optical image of a freestanding 3 nm‐thick gold film transferred onto a TEM support grid with 30 µm‐diameter windows. i, HR‐TEM image of the crystalline structure and an enlarged image of the selected area, showing an interplanar spacing of ≈0.24 nm for the 3 nm‐thick gold film.

**FIGURE 3:**
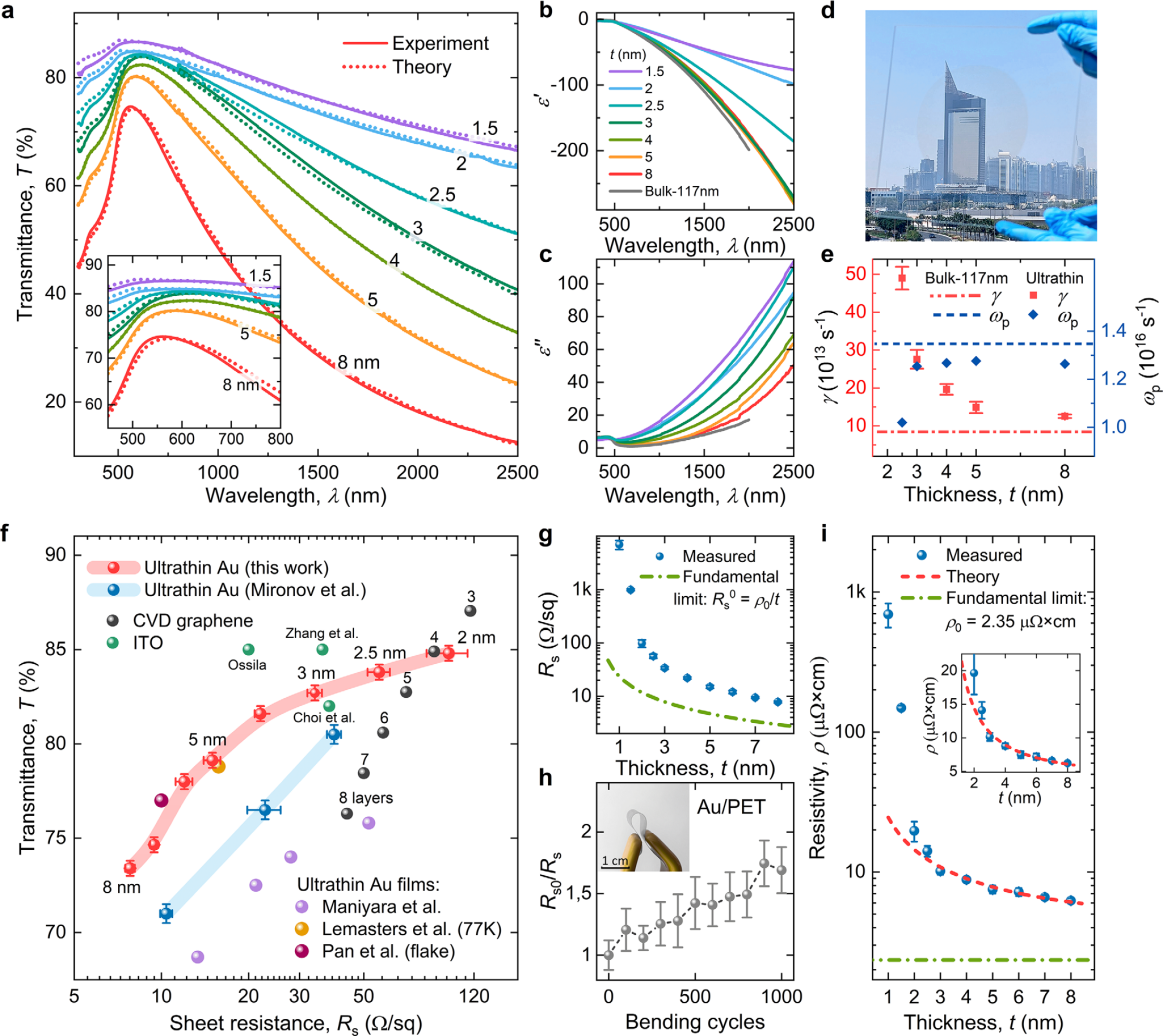
Optoelectronic properties of ultrathin gold. a, Optical properties of ultrathin gold films transferred onto a transparent substrate. Measured total transmittance spectra of gold films on CaF_2_ for various film thicknesses (solid lines) compared with calculated transmittance spectra (dashed lines), derived from ellipsometrically measured optical constants. b, Real *ε*’ and c, imaginary *ε*″ parts of the dielectric permittivity extracted from spectroscopic ellipsometry measurements of gold films on CaF_2_. d, Photograph taken through a 5 nm‐thick gold film transferred onto glass, showing its high transparency. e, Drude model parameters (*ω*_p_ and *γ*) obtained from ellipsometric analysis, in comparison with parameters for a 117 nm‐thick bulk gold film. f, Optoelectronic figure‐of‐merit of transparent conductive films: sheet resistance and transmittance at *λ* = 550 nm for the synthesized ultrathin gold films in comparison with previously reported ultrathin gold, ITO films, and multilayered CVD‐graphene. g, Measured sheet resistance of ultrathin gold films as a function of thickness compared to the theoretical minimal limit for bulk crystalline gold. h, Changes in sheet resistance of a 5 nm‐thick gold film upon repeated bending cycles. The inset shows the bending configuration on PET. i, Resistivity of ultrathin gold films with fitting based on a theoretical thickness‐dependent model, compared to the fundamental limit of resistivity for bulk crystalline gold.

**FIGURE 4:**
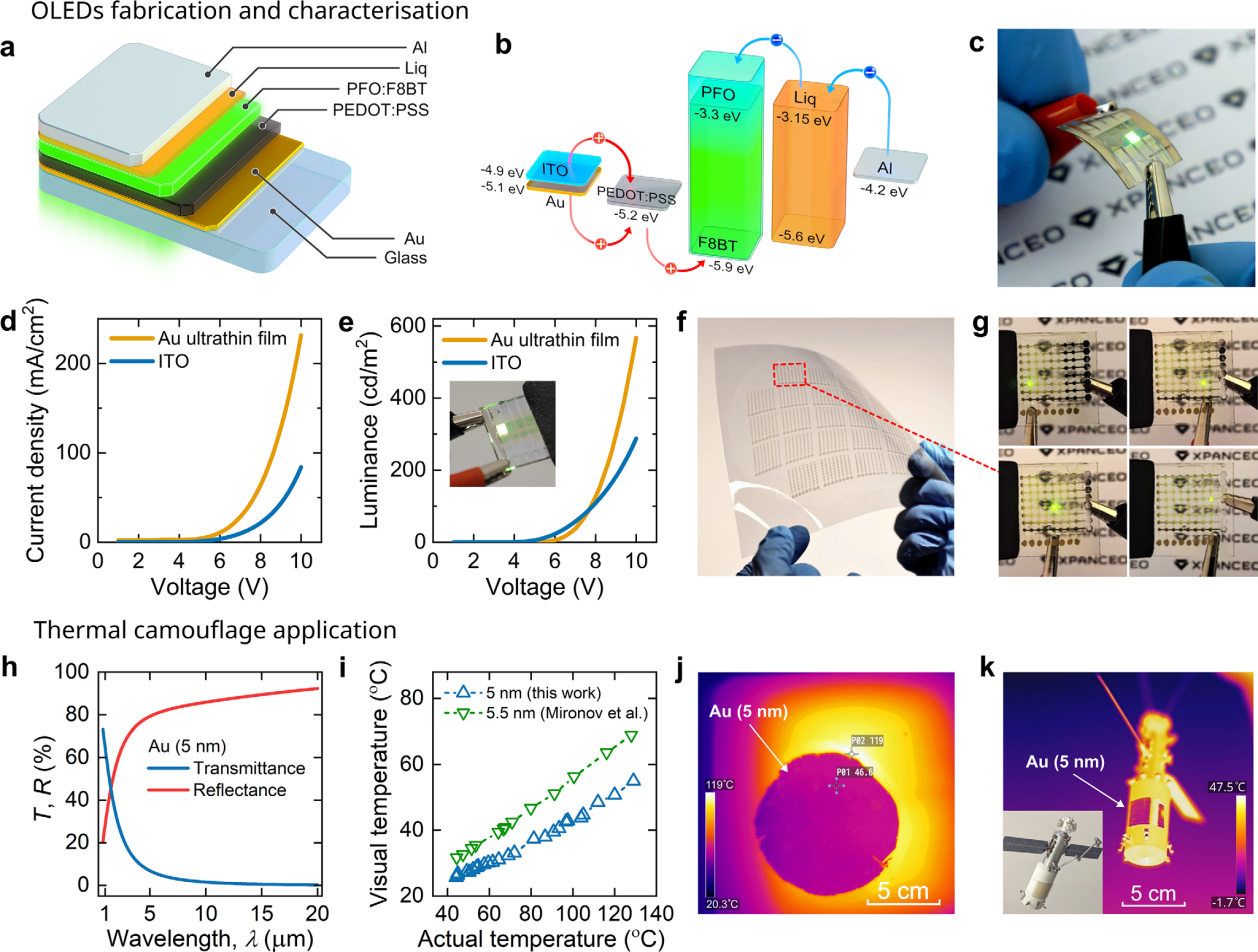
Optoelectronics and thermal management of ultrathin gold. a, OLED device schematics with ultrathin gold as an anode. b, OLED energy band diagram comparison of gold and ITO as an anode. c, Photo of flexible OLED with a gold anode on PET. d, Current density and e, luminance of OLEDs on glass substrate based on 8 nm‐thick gold film and ITO anodes, as a function of applied voltage (Inset: photo of ultrathin gold‐based OLED on a glass substrate). f, Photos of anode arrays for OLED on PET with g, addressable illuminated pixel on a 6‐inch scale anode. h, Transfer matrix‐calculated transmittance and reflectance spectra of 5 nm‐thick gold on CaF_2_. i, The dependence of the thermal camera's visual temperature of a 5 nm‐thick gold film on its actual temperature. For comparison, we also added the previous results [4] of 5.5 nm‐thick gold film thermal camouflage. j, Thermal camera image of a 4‐inch gold film on PET substrate covering a hot plate with the background temperature set at 120 °C. k, Thermal camera image of a satellite covered by a 5 nm‐thick ultrathin gold. The inset shows an image of a satellite.

**FIGURE 5:**
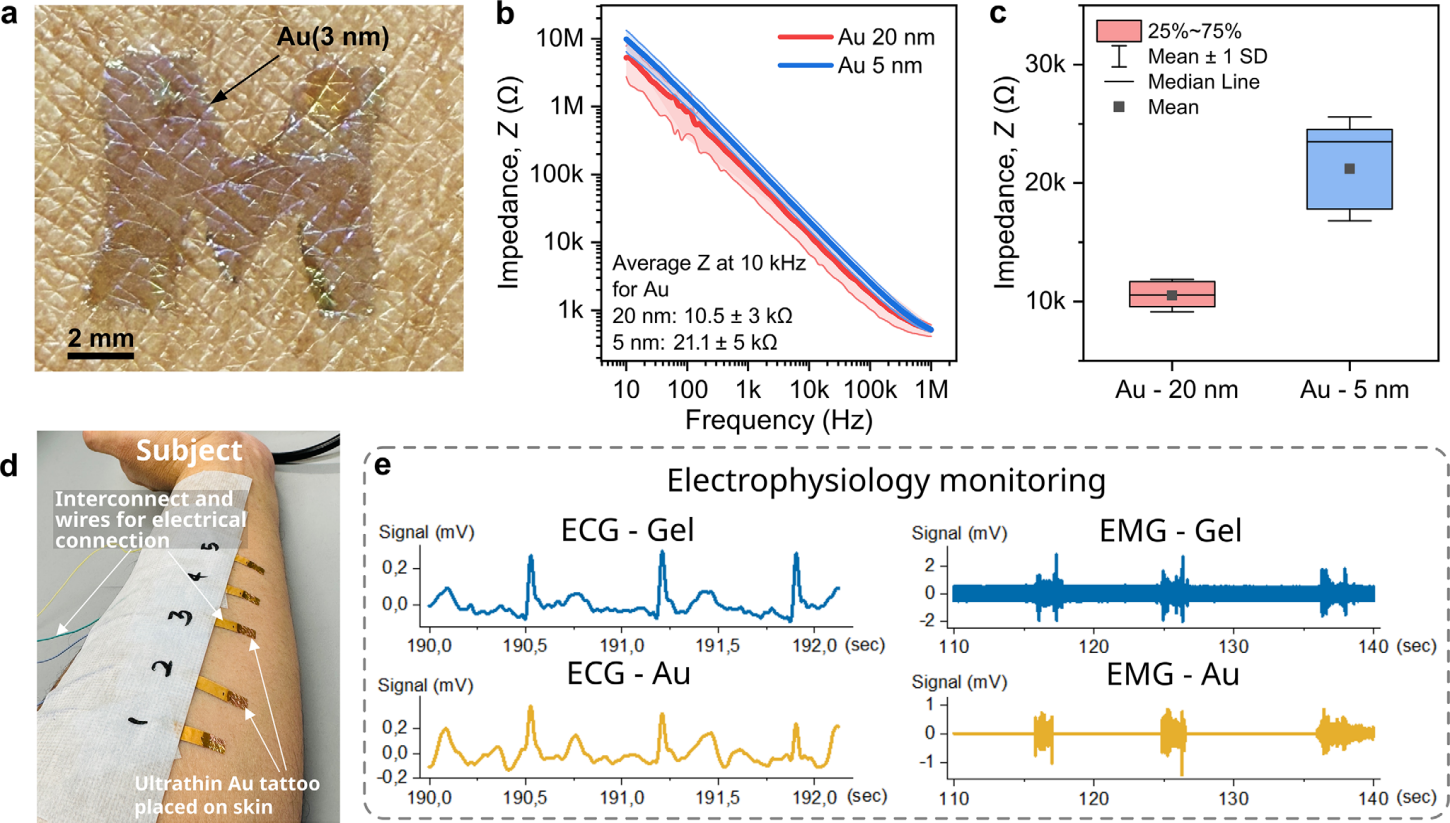
Ultrathin gold biosensors. a, Photo of ultrathin Au tattoo in the form of “M” letter transferred onto a human forearm. b, Electrode‐skin impedance characterization in a broad frequency range for ultrathin Au films with thicknesses of 5 nm and 20 nm, after transfer to skin (N = 5 electrodes). c, The mean impedance values (at 10 kHz) and their statistical distribution for the 5 nm thick (blue) and 20 nm thick (red) Au tattoo electrodes. d, Photo of ultrathin Au tattoos array transferred onto a human forearm (square of individual patch is 5 × 8 mm^2^) for tattoo‐skin impedance measurement setup. e, Results of electrophysiology monitoring of ECG and EMG, comparing two types of electrodes: Au ultrathin tattoos and commercial Ag/AgCl gel electrodes in the band between 1–30 Hz for ECG and 40–120 Hz for EMG.

Inspired by the chemical vapor deposition (CVD) graphene growth and transfer method shown in Figure 1a, our ultrathin gold fabrication process involves three key stages, presented in Figure 1b. The first step is the preparation of an atomically smooth copper foil via a template stripping method, shown in Figure 1b‐1 and Figure 11b‐2. In contrast to our previous route, where the copper growth surface was an electron‐beam‐evaporated layer whose roughness was set by its columnar grain structure [4], the copper foil here is a free‐standing template‐stripped substrate whose gold‐facing surface inherits the atomic flatness of the underlying single‐crystal Si(111) wafer. The second step is the physical vapor deposition of an ultrathin gold layer (Figure 1b‐3) and a PMMA coating layer. Finally, we etch the Cu foil (Figure 1b‐4) and transfer the ultrathin gold film on the target substrate (Figure 1b‐5 and Figure 1b‐6). The technological relevance of this synthesis method (Figure 1b) is underscored by its inherent stability. For example, we demonstrate this by fabricating continuous 5 nm‐thick gold films on 6‐inch silicon wafers and subsequently transferring them onto flexible polyethylene terephthalate (PET) substrates, highlighting the method's potential for industrial‐scale manufacturing and integration into flexible optoelectronics (Figure 1c).

The success of our method is rooted in overcoming a fundamental thermodynamic barrier that typically drives metals toward three‐dimensional (3D) island formation (Volmer‐Weber growth) [18]. Our approach utilizes the metal‐substrate energetics to favor a layer‐by‐layer (2D) Frank‐van der Merwe growth regime. As illustrated by the energy diagram in Figure 1d, the choice of copper as a growth template is critical. The adsorption energy of gold adatoms on copper (*E*_ads_ ≈ 7.78 J/m^2^) is close to the cohesive energy of gold itself, satisfying the thermodynamic criterion for 2D growth–*E*_ads_ ≫ 2*γ*_m_, where *γ*_m_ ≈ 1.12 J/m^2^ is the surface free energy of gold [10, 12, 23, 24, 25]. In contrast, substrates like SiO_2_ exhibit low adsorption energy, which invariably leads to 3D islanding of gold (Figure 1d).

To gain direct atomic‐scale insights into this thermodynamically‐driven process, we performed molecular dynamics (MD) simulations of gold deposition (Figure 1e) [26]. The simulations starkly contrast the growth outcomes on different surfaces. On the copper template (Figure 1e), gold atoms self‐assemble into a uniform, continuous, and highly crystalline monolayer, perfectly replicating the Frank‐van der Merwe mechanism suggested by Figure 1d. This occurs even under anisotropic deposition conditions, where Au atoms are deposited exclusively on one half of the substrate surface, designed to represent a worst‐case scenario (for MD details see Note S1 and Methods section). Conversely, gold deposition on a silicon surface (Figure 1e) results in the formation of disordered, discrete islands, a clear signature of the 3D Volmer‐Weber growth mechanism. These results provide powerful atomic‐scale validation of our choice of copper as the ideal growth template.

In addition, we directly visualize the morphology of 3 nm‐thick gold films grown on different substrates using high‐resolution scanning electron microscopy (HR‐SEM) in Figure 1f. Figure 1f provides a clear confirmation of the distinct growth mechanisms anticipated from Figure 1d,e. Gold grown on our template‐stripped copper foil and transferred to a SiO_2_/Si substrate yields a continuous, featureless film, demonstrating the successful realization of 2D growth. In stark contrast, gold deposited directly on an exfoliated MoS_2_ crystal produces a percolated network [15], while growth on a bare SiO_2_/Si substrate results in the classic, discontinuous island‐like morphology characteristic of 3D growth. This substrate‐dependent difference in film quality unequivocally demonstrates that our technology in Figure 1b enables the synthesis of continuous atomically‐thin and transferrable gold films.

### 2.2 Atomic Smoothness of Fabricated Ultrathin Gold

The success of our fabrication method in Figure 1b strongly depends on the morphology of copper foil surface after the step in Figure 1b‐2 since the deposited gold layer repeats a copper relief. HR‐SEM of copper in Figure 2a reveals a uniform and featureless surface. We further quantify this high‐quality surface using atomic force microscopy (AFM), which shows atomically flat terraces in Figure 2b. Over a 4 × 4 µm^2^ area (Figure 2b), we measure a root‐mean‐square (RMS) roughness of just 0.24 nm, a value that decreases to 0.16 nm at higher magnification in Figure 2c. This is markedly smoother and more crystalline, with fewer grain boundaries, than the evaporated copper used in our earlier work [4], where the grain structure of the deposited metal imposed a hard lower bound of 3.5 nm on the continuous gold thickness (see the side‐by‐side comparison in Note S2). Commercial rolled copper foil, including graphene‐CVD grade, is rougher still, since its surface is dominated by rolling waviness with roughness of order tens of nanometres, and it therefore cannot serve as a template for continuous gold at the 1.5 to 2 nm thicknesses reached here, irrespective of the polishing protocol (Note S2). Since the deposited gold replicates the copper relief, and since at a few nanometres even sub‐nanometre template corrugation becomes comparable to the film thickness, this suppression of template roughness is the decisive factor enabling continuous gold down to 1.5 nm and the corresponding gain in optical transmittance. This level of smoothness provides a uniform energetic landscape for subsequent layer‐by‐layer gold growth.

Having established the quality of copper template, we next looked for direct evidence that it suppresses the 3D Volmer‐Weber islands formation typical for ultrathin metals. For this purpose, we deposited a nominal 1 nm‐thick gold film–a regime where island formation is most pronounced on conventional substrates–and reexamined the surface. Strikingly, AFM image in Figure 2d shows no formation of discrete islands since the surface morphology and RMS roughness of 0.18 nm remain almost unchanged from the bare copper in Figure 2c with 0.16 nm RMS roughness. This provides additional evidence of a continuous, layer‐by‐layer Frank‐van der Merwe growth mode, confirming that our engineered substrate overcomes the thermodynamic barrier to assist 2D metal growth.

The ultimate validation of our fabrication process (Figure 1b) lies in the structural integrity of ultrathin gold films after transfer to a SiO_2_/Si substrate. Across a range of thicknesses from 2 to 8 nm, HR‐SEM imaging confirms the gold films are continuous and uniform, with only rare nanoscale pinholes observed in the thinnest 2 nm film (Figure 2e). This visual evidence is corroborated by AFM, which measures an atomic surface smoothness with RMS roughness below 0.4 nm for all considered thicknesses (Figure 2f). This demonstrates that the atomic‐scale quality of the copper template is faithfully preserved in the final transferred ultrathin gold films. AFM step‐height measurements across cracks and edges confirm the nominal film thicknesses (Figure 2g and Note S2). The mechanical robustness of these ultrathin gold films is highlighted by our ability to create freestanding membranes suspended over a TEM grid (Figure 2h), emphasizing their immediate utility for device integration.

Finally, to resolve the film's atomic structure, we performed high‐resolution transmission electron microscopy (HR‐TEM) on a suspended 3 nm‐thick film. The images reveal a polycrystalline structure composed of large, highly‐ordered crystalline domains (Figure 2i). Crucially, the lateral dimensions of these single‐crystal grains, often exceeding 20 nm, are several times larger than the film thickness of 3 nm, confirming the quasi‐2D nature of synthesized gold. Magnified analyses of these domains in Figure 2i shows the characteristic period lattice fringes of gold atoms with an interplanar spacing of ≈0.24 nm, traditionally assigned to the close‐packed (111) orientation [34]. The summarized in Figure 2 atomic‐level analysis provides a definitive confirmation of the high structural quality of our ultrathin gold films.

### 2.3 Performance of Ultrathin Gold Transparent Electrodes

We next quantify the optoelectronic and mechanical properties of our ultrathin gold films that are paramount for their technological applications. The optical properties of ultrathin gold films, transferred onto transparent CaF_2_ substrates, were first characterized in the broad spectral range (300–2500 nm). The resulting total transmittance spectra for films with thickness from 1.5 to 8 nm are presented in Figure 3a. The spectra exhibit the expected metallic optical response, in which transmittance decreases at longer wavelengths due to free carrier reflectance screening, a characteristic that persists even for the thinnest films of 1.5 and 2 nm. As film thickness decreases, the peak transmittance demonstrates a monotonous increase, rising from 74.6% for the 8 nm film to 86.6% for the 1.5 nm film (Figure 3a). This monotonic gain, and in particular the access to the thinnest continuous films, is a direct consequence of the reduced template roughness described above, which suppresses the scattering and parasitic absorption associated with morphological discontinuities in earlier rougher films [4]. These values rank among the highest reported [11, 35, 36] for ultrathin gold films of comparable thicknesses (Figure 3f).

To better understand the fundamental optical response of our ultrathin gold, we determined the film complex dielectric function ε = ε′ + *i*ε″, using spectroscopic ellipsometry over the same spectral range. The real (ε′) and imaginary (ε′′) parts of the dielectric function, extracted from point‐by‐point fit of the ellipsometric parameters (see Methods), are plotted in Figure 3b,c, respectively, alongside reference data for a bulk gold film [37]. For all thicknesses, ε′ < 0, confirming a pronounced metallic optical response. Crucially, for films from 3 to 8 nm, the spectral dependence of ε′ closely follows that of bulk gold. This remarkable correspondence is a direct signature of the film's high crystalline quality and continuity. It indicates a suppression of non‐ideal optical responses, such as localized surface plasmon resonances, that are typically observed in ultrathin metal films plagued by nanoscopic discontinuities or island‐like morphologies. To validate our ellipsometric analysis, we calculated the transmittance spectra using the extracted dielectric functions via the transfer‐matrix method [38]. The calculated spectra (Figure 3a) show excellent agreement with the measured transmittance data across the entire wavelength range, confirming the accuracy of our optical characterization. Moreover, the high optical quality and structural integrity are further demonstrated at the wafer scale, as shown by a photo in Figure 3d taken through a 5 nm‐thick film with 6‐inch dimensions on a glass revealing gold high visual transparency.

Further analysis based on the Drude oscillator model term, ε = –$\omega_{p}^{2}$/(ω^2^ + *i*ωγ), where ω is the light frequency, allowed us to extract the plasma frequency (ω_*p*_) and the damping factor (γ). The fitted values, compared to bulk gold in Figure 3e, reveal ω_*p*_ = (1.27 ± 0.02)·10^16^ s^−1^ for 3–8 nm‐thick films, which is within 6% of the bulk value (ω_*p*_ = 1.35·10^16^ s^−1^). Since ω_*p*_ is directly proportional to the square root (ω_*p*_∝ *n^1/2^*) of the free carrier density (*n*), this near‐bulk value provides quantitative evidence that our fabrication method yields ultrathin gold films with a free electron density close to that of high purity crystalline gold, indicating high electronic quality with minimal defect density. Conversely, the damping factor γ, which represents optical losses, increases as the film thickness decreases (Figure 3e). This trend is consistent with the expected increase in electron scattering from the film surfaces, which becomes a dominant loss mechanism as the film thickness approaches the electron mean free path [37].

The high quality of our ultrathin gold is further confirmed by electrical measurements. The measured sheet resistance (Figure 3g) and calculated resistivity (Figure 3i) both decrease with increasing thickness, closely approaching the theoretical minimal limits for bulk crystalline gold. This proximity to the fundamental limit provides evidence that electron scattering at grain boundaries is minimized, reinforcing the conclusion from microscopic studies in Figure 2 of a high‐quality polycrystalline structure composed of large, well‐connected crystalline domains. To assess the technological potential of these films as transparent conductors, we benchmark their performance using the standard figure‐of‐merit, plotting transmittance at *λ* = 550 nm against sheet resistance in Figure 3f. Our ultrathin gold films occupy a promising space in Figure 3f, demonstrating a trade‐off between transparency and conductivity that is superior to previously reported ultrathin gold [4, 11, 35, 36] and multilayered CVD graphene [39, 40] and being competitive with the industry‐standard indium tin oxide (ITO) [41, 42]. This exceptional performance stems directly from the film's continuous high‐quality morphology and atomic smoothness (Figure 2), which circumvents the percolation threshold limitations that typically degrade the conductivity of metallic films in the sub‐10 nm regime.

It is worth noting that the films exhibit electrical transport characteristics governed by their reduced dimensionality (Figure 3i). The measured resistivity is substantially higher than the fundamental limit for bulk single‐crystalline gold (ρ_0_) due to additional electron scattering mechanisms that become dominant at the nanoscale. This phenomenon is well‐described by a total resistivity model, ρ  =  ρ_0_ + ρ_*s*_ + ρ_*g*_, which sums the contributions from intrinsic bulk scattering (ρ_0_), electron‐surface scattering (ρ_*s*_), and grain‐boundary scattering (ρ_*g*_). These size effects are particularly pronounced in our ultrathin films, where the thickness (*t*) is inherently much smaller than the electron mean free path in bulk gold (Λ ≈ 40 nm). The thickness‐dependent resistivity of such polycrystalline films is quantitatively captured by a model combining the Fuchs‐Sondheimer (FS) and Mayadas‐Shatzkes (MS) formalisms [43]. In the limit where *t* ≪ Λ, this model simplifies to [44]:

(1) $$\begin{array}{ccc} \rho & = & \rho_{0}\;\times \frac{1-p}{1+p}\times {\left(\frac{t}{\Lambda }ln\left(\frac{\Lambda }{t}\right)\right)}^{-1}\\ & & +\;\rho_{0}\left[{\left(1-\frac{3}{2}\alpha +3\alpha^{2}-3\alpha^{3}ln\left(1+\frac{1}{\alpha }\right)\right)}^{-1}-1\right] \end{array}$$

where the parameter α = (Λ/*D*)·(*R*/(1–*R*)) depends on the mean grain size (*D* = *a* × *t*, with the phenomenological proportionality constant *a* = 10 consistent with the grain dimensions resolved by HR‐TEM in Figure 2i), the phenomenological coefficients *p* and *R* represents the fraction of electrons scattered specularly at the surfaces and the reflection coefficient at grain boundaries, respectively. As seen from Figure 3i, this model provides an excellent agreement with the measured data for films in the 2–8 nm range, with physically reasonable parameters *p* = 0.29 and *R* = 0.31. These values align with previously reported results for continuous thin gold films [37, 45], confirming that the electrical transport in our ultrathin gold is dominated by surface‐ and grain‐boundary limit scattering.

The thickness dependence of transmittance and sheet resistance (Figure 3f,g) defines a trade‐off that guides the choice of film thickness for a given application. Films below 5 nm offer the highest visible transmittance, reaching a peak of 86.6% for the 1.5 nm film, but at the cost of a higher sheet resistance on the order of 100 Ω/□ for the 2 nm film. Films of 5 to 8 nm instead combine a moderate transmittance of 79.1% and 73.4% at λ = 550 nm with a low sheet resistance of 14.9 and 7.8 Ω/□, respectively, comparable to commercial ITO. Accordingly, the demonstrator devices in the following sections use the thickness best matched to each function.

Another critical requirement for flexible electronics is mechanical robustness of transparent electrodes. We evaluate the durability of our ultrathin gold films by subjecting a 5 nm‐thick film on a flexible PET substrate to 1000 repeated bending cycles under extreme bending radius of ≈3 mm (inset in Figure 3h). Figure 3h shows that the sheet resistance exhibits only a minimal increase, demonstrating resilience to mechanical fatigue. This mechanical stability (Figure 3h) and superior optoelectronic properties validates the suitability of our ultrathin gold as a foundational platform for the high‐performance flexible optoelectronic and bio‐integrated systems demonstrated in the subsequent sections.

### 2.4 Electronics and Photonics of Ultrathin Gold Films

The combination of high electrical conductivity, optical transparency, and mechanical flexibility (Figure 3) in our ultrathin gold positions it as a compelling alternative to brittle indium tin oxide (ITO) electrodes [10]. To demonstrate this potential, an 8 nm‐thick ultrathin gold film (*R*_s_ = 7.8 Ω/□, comparable to the commercial ITO reference; see Figure 3f) was patterned and integrated as the transparent anode in a solution‐processed organic light‐emitting diodes (OLEDs). The device architecture (Figure 4a) consists of a multi‐layer organic stack sequentially deposited onto the gold anode with the band diagram shown in Figure 4b and a device photo provided in Figure 4c. A quantitative comparison in Figure 4d,e against a control device using a commercial ITO anode (see Methods and Note S3) reveals that at equivalent applied voltages, the gold‐based devices exhibit higher current densities and luminance. This performance gain is likely attributed to more efficient charge carrier injection from the gold to a PEDOT:PSS hole‐injection layer with an energy barrier of 0.1 eV compared to 0.3 eV for ITO, as seen from the band diagram in Figure 4b. Beyond this favorable injection barrier, the atomic smoothness of the transferred anode (RMS = 0.38 nm for the 8 nm film, Figure 2f) is itself a prerequisite for OLED operation. The organic stack separating anode and cathode is only ≈80 to 120 nm thick, with individual transport and injection layers about 10 nm. Therefore, protrusions on a rougher electrode could shunt the stack and cause local breakdown, whereas the present atomically smooth gold anode removes this shunting mechanism. Moreover, Figure 4c shows that our OLEDs maintain stable and uniform light emission even at bending condition. For scalability demonstration, we fabricated a large‐scale, matrix‐addressable, and transparent 8×8 pixel OLED display using a 6‐inch scale multy‐anode array on a flexible PET substrate, presented in Figure 4f,g. This combination of optoelectronic performance, mechanical robustness, and manufacturing scalability establishes our fabricated ultrathin gold as a perspective platform for flexible displays.

Beyond optoelectronics, the gold films’ optical response enables applications in thermal management. Indeed, effective thermal camouflage requires two critical properties: high transparency in the visible spectrum to maintain the object's visual appearance, and low emissivity in the thermal infrared range to suppress the object's heat signature [6]. Our ultrathin gold films satisfy both conditions: they exhibit high visible transmittance (Figure 3a), while their low infrared emissivity is a direct consequence of high metallic reflectivity, which minimizes thermal absorption *α*_λ_ and emission *ε*_λ_ as dictated by Kirchoff's law (*α*_λ_ Figure 4 = *ε*_λ_) of thermal radiation. This behavior is confirmed by transmittance and reflectance spectra in near‐ and mid‐infrared (Figure 4h). As a proof‐of‐concept, we measured the visual temperatures of heated surfaces covered with a 5 nm‐thick gold film using a thermal infrared camera (see Methods). The results in Figure 4I,j show a linear dependence of the measured visual temperature on the actual background temperature with a slope of ≈0.32, confirming effective camouflaging. Relative to the rougher films of our earlier work [4] (RMS ≈ 2 nm), the present films (RMS < 0.4 nm) lowers the apparent visual temperature by ≈30% at the same actual temperature (Figure 4i). This improvement originates from the better optical properties and the reduced surface roughness with the suppressed scattering and parasitic absorption, which by Kirchhoff's law lowers the infrared emission that sets the thermal signature. Moreover, the gold film's utility for non‐planar objects is shown in Figure 4k, where a transferred Au/PDMS film conceals a satellite‐shaped toy from a thermal infrared camera, while a corresponding optical photograph confirms the film's high visible range transparency. These examples link our ultrathin gold films with thermal management applications.

### 2.5 High‐Fidelity Epidermal Electrophysiology With Ultrathin Gold Tattoos

Apart from optoelectronics applications (Figure 4), our ultrathin gold films can be used in wearable devices, such as electronic tattoos for electrophysiological monitoring [9, 46]. To demonstrate this potential, we prepared arrays of electrodes of ultrathin gold tattoos with 5 nm and 20 nm thicknesses with a 5 × 8 mm^2^ square patch (see Methods and Note S4). These thicknesses were selected for adequate trace conductivity and mechanical robustness over repeated deformation, since the sensor performance is governed by the electrode‐skin interface rather than by the film sheet resistance. The electrodes were transferred onto the human forearm, demonstrating their suitability as wearable devices capable of maintaining stable and conformal skin contact (Figure 5a). The devices remained mechanically intact on the skin and exhibited consistent electrical performance, with impedance remaining stable across multiple measurements (with *N* = 5 electrodes). Figures 5b‐c shows the evaluation of the electrophysiological characteristics of these electrodes, including impedance measurements between the tattoo and the skin across a broad frequency range (10 Hz–1 MHz). The gold e‐tattoo impedance (Figure 5b) exhibits a representative exponential decrease with increasing frequency, which is characteristic of the capacitive coupling at the electrode‐skin interface. Using the standard 10 kHz frequency as a figure‐of‐merit for performance, we determined the mean impedance values and their statistical distribution, shown in Figure 5c. The 20 nm‐thick gold tattoos exhibit an average impedance of 10.5 ± 3.0 kΩ, while the thinner 5 nm tattoos show a higher impedance of 20.3 ± 6.0 kΩ (Figure 5c). This thickness‐dependent behavior highlights a fundamental design trade‐off, where the enhanced flexibility of thinner films is balanced against their inherently higher electrical resistance. Critically, these impedance characteristics are comparable with those of state‐of‐the‐art multilayer graphene‐based electronic tattoos with impedance values of 7–20 kΩ, measured under identical conditions [9, 47]. Next, we implement gold tattoos to measure heart activity (electrocardiogram, ECG) and muscle activity (electromyogram, EMG), with the experimental setup shown in Figure 5d. The signal quality of ECG and EMG (Figure 5e) was quantified by the signal‐to‐noise ratio (SNR) in the frequency domain and benchmarked against commercial Ag/AgCl gel electrodes, the clinical “gold standard”. For ECG monitoring, the gold electrodes achieve SNR = 14.9 dB, significantly higher than SNR = 12.7 dB recorded for the gel electrode. Similarly, for EMG monitoring, the gold electrodes’ SNR = 69.5 dB surpasses the gel electrodes’ SNR = 62.4 dB. This enhanced performance occurs despite the gold electrode exhibiting higher baseline fluctuations due to its greater electrode‐to‐skin impedance. This outcome demonstrates that for epidermal sensing, the quality of the mechanical coupling at the skin interface is a more important factor for signal fidelity than the intrinsic impedance alone. The exceptional conformability of the ultrathin gold films ensures a stable, intimate contact that minimizes motion artefacts and improves the transduction of the biological signal, thereby boosting the SNR. Collectively, these tests confirm that our ultrathin gold electrodes could provide an effective platform for electrophysiological sensing, capable of capturing high‐quality signals that outperform traditional gel electrodes.

## 3 Conclusions and Outlook

Our work introduces a technological platform for producing wafer‐scale, transferable, and continuous ultrathin gold films with thicknesses approaching the atomic limit. By merging template stripping [22] with a graphene‐inspired transfer technique [4], we overcome the fundamental thermodynamics barriers that typically lead to discontinuous island growth. The resulting films exhibit a combination of atomic smoothness, near‐bulk electronic quality, high optoelectronic performance rivaling ITO [41, 42] and graphene [40], and robust mechanical flexibility. We have validated the technological readiness of synthesized ultrathin gold by demonstrating high‐performance flexible OLEDs, thermal camouflage, and skin‐interfaces for medical diagnostics. In addition, the principles of our method are easily generalizable, paving the way for a new materials library of quasi‐2D metals beyond gold, including silver, palladium, and platinum. Still, the key challenge is pushing this technology toward 1 nm‐thick metallic films, for example, by employing alternative to PMMA stabilizing interfaces. Among them sulfur‐based materials like MoS_2_ [15] and ZnS [48] are the most promising candidates since they have high adsorption energy to gold atoms. Further performance enhancements in conductivity, transparency, and flexibility can be achieved by advancing our technique to produce monocrystalline films, for instance, by using single‐crystal copper templates [21], thereby eliminating the grain boundary scattering and unlocking the ultrathin gold full potential.

### 3.1 Human Subject

All human subject experiments in this study were conducted in accordance with institutional guidelines. This study was reviewed by the University of Massachusetts Amherst Institutional Review Board and was determined to be exempt from IRB approval, as it involved non‐invasive electrophysiological measurements on healthy adult volunteers with no collection of identifiable private information under HRPO Determination Number: 7989.

## 4 Experimental Methods

### 4.1 Fabrication and Transfer of Au Films

First, for the preparation of ultrasmooth copper foil for the gold growth, a 500 nm‐thick copper layer was deposited onto a silicon (111) wafer, using high‐purity pellets (99.999% Cu from Kurt J. Lesker Co) by electron‐beam evaporation in a high vacuum setup (Nano‐Master NEE‐4000) with a base pressure of approximately 10^−7^−10^−6^ Torr at a rate of ∼10 Å/s. After deposition of the initial copper layer, the copper foil was electroplated to increase its thickness by electrochemical deposition in a copper acid electrolyte, so that the foil could be self‐supporting, with a typical thickness of 30−60 µm, which can vary. For the preparation of the electrolyte, sulfuric acid, copper sulfate pentahydrate, and water were used. Sodium chloride (10‐20 mg/L in the prepared solution) was added as an electrolyte stabilizer to improve coating uniformity. Then, before gold deposition, copper foil was peeled from the silicon (111) wafer with tweezers (Figure 1a‐3) and, as a substrate, immediately placed in a high‐vacuum e‐beam evaporator.

Subsequently, gold films of 1−8 nm were deposited on the surface of the copper foil at a rate of 0.3−0.5 Å/s in an electron‐beam evaporation system and at room temperature with a base pressure of approximately 8·10^−7^ Torr. Deposition parameters, such as growth rate and mass‐equivalent thicknesses, were controlled in situ by a calibrated quartz crystal thickness monitor, which provides high reproducibility of gold film thickness.

Following this, the prepared layered Au/Cu structure was coated with a protective layer of polymethyl methacrylate (PMMA) by spin coating. This PMMA layer is necessary to preserve the films during transfer and serves a function analogous to an intermediate supporting layer, as used, for example, in the graphene transfer process. After the thick copper layer beneath the gold films was completely dissolved in ammonium persulfate, a freestanding PMMA/Au film was obtained. The gold films were then transferred to glass by “scooping” the PMMA/Au stack from deionized water.

Finally, the protective PMMA layer was carefully removed. For this, the Au/glass samples were cleaned in acetone, followed by rinsing in isopropanol and drying with a stream of nitrogen. In the case of film deposition on a PET surface, toluene was used instead of acetone for PMMA removal.

### 4.2 Surface Morphology Characterization

The surface morphology and thickness of ultrathin Au films were characterized using an atomic force microscope (AFM, NT‐MDT Spectrum Instruments, Ntegra II) operating in the Hybrid (peak‐force) mode. AFM scans were conducted at 400 × 400 pixels using ScanSens (NSG10) cantilever tips with a spring constant of 3.5 N/m, a tip radius of <10 nm, and a resonant frequency of 180 kHz. Gwyddion software was used to analyze the scans and determine the root‐mean‐square roughness.

To visualize the surface coverage and homogeneity of the fabricated ultrathin Au films with high accuracy the high‐resolution scanning electron microscope (HR‐SEM, Helios G4 PFIB, Thermo Fisher Scientific) was used operating with an acceleration voltage of 2 – 5 kV and a current of 25 pA, with a working distance of 2 – 4 mm, providing up to 1 000 000x magnification with scan dimensions of 3072 × 2188 pixels. In some cases, a standard SEM (JEOL JSM7001F) was used to visualize the surface morphology of the ultrathin films. SEM imaging was performed with an acceleration voltage of 30 kV and a current of 67 µA, capturing scans of 1960 × 1280 pixels across various areas.

### 4.3 Optical and Electrical Properties Characterization

Optical absolute transmittance spectra of ultrathin Au films transferred onto CaF_2_ were measured with a spectrophotometer (Cary 5000 UV‐Vis‐NIR, Agilent Tech.) over the wavelength range of 300–2500 nm. To ensure representativeness, transmittance was collected and averaged from 5 different areas on the film.

The dielectric permittivity of the transferred ultrathin Au films was determined using a variable‐angle spectroscopic ellipsometer (VASE, J.A. Woollam Co.). Measurements were carried out in transmission mode, with incidence angles ranging from 50° to 60° in 5° increments, and a broad spectral range of 300 to 2500 nm with a 1 nm step. To accurately fit the measured ellipsometric parameters *Ψ* and *Δ*, a layered model and point‐by‐point fitting method [49] were employed to determine the effective dielectric function *ε* = *ε*’ + i*ε*″ at each spectral point.

Sheet resistances *R*_s_ of the ultrathin gold films were measured using a Jandel RM3000 four‐point probe system with probe tip radii of 40 µm and a probe spacing of 0.6 mm. Measurements were taken and averaged from 8–10 different film locations.

### 4.4 Mechanical Flexibility Study

For the bending experiment, Au films of different thicknesses were transferred onto flexible PET (polyethylene terephthalate) stripes. Bending tests were performed using a home‐built setup; the bending radius was around 2.5 mm, and the rate of the bending cycles was about 60 times per minute.

### 4.5 Thermal Camouflage Measurements

The analysis of the thermal camouflaging effect provided by ultrathin gold films was conducted using an InfiRay T3Pro thermal IR camera. For a comprehensive investigation, various types of samples were prepared and measured. A thick gold film was transferred onto PET substrates, and the Au/PET samples were placed on a hot plate and heated to 150 °C. During the controlled increase of the sample's actual temperature, the IR camera measured its visible temperature. Similarly, a 5 nm‐thick gold film was applied to 4‐inch PET sheets, and the visual temperatures of these heated Au/PET sheets were also measured using the thermal IR camera to assess their camouflaging properties. To demonstrate the method's versatility, a 5 nm‐thick gold film was transferred onto the surface of a glass beaker. Subsequently, the beaker with the transferred film was filled with water and heated to 70 °C, which was used to evaluate the effectiveness of camouflaging on curved surfaces under conditions simulating real‐world scenarios, while maintaining optical transparency. Throughout all experiments, as the sample's actual temperature increased, the thermal IR camera (Torus, Handheld Thermal Infrared Imager, SC600‐A) recorded the corresponding visible temperature, allowing a quantitative assessment of the thermal camouflage effect.

### 4.6 Fabrication of OLEDs

#### 4.6.1 Materials

ITO glass substrates (20 Ω/sq, thickness 1.1 mm), poly(9,9‐di‐n‐octylfluorenyl‐2,7‐diyl) PFO (Mw = 260 817), poly(3,4‐ethylenedioxythiophene) polystyrene sulfonate PEDOT PSS (Clevios P VP AI 4083), 8‐hydroxyquinolinolato‐lithium Liq were purchased from Ossila. Poly(9,9‐dioctylfluorene‐alt‐benzothiadiazole) F8BT (Mw > 20 000) was purchased from Lumtec. PFO and F8BT were dissolved in toluene (PFO:F8BT = 95:5, overall concentration 10 mg/ml). Liq was dissolved in isopropyl alcohol (1 mg/ml). All solutions were previously filtered by 0.45 µm PTFE syringe filters.

#### 4.6.2 Device Fabrication

ITO glass substrates (20 Ω/sq, thickness 1.1 mm, Ossila) were subsequently cleaned in the ultrasonic bath with acetone (5 min) and isopropanol (5 min). ITO substrates and substrates coated with gold films (glass 1.1 mm thick, PET 0.1 mm thick) were treated in UV Ozone cleaner (Ossila) for 10 min prior to the coating of the PEDOT:PSS layer and placed in the glove box (Ossila) with N_2_ atmosphere. All substrates were subsequently coated with PEDOT:PSS, PFO:F8BT, and Liq layers by a spin coater (Ossila) and baked on a hot plate at 90 °C for 10 min after each coating step. After that, substrates were removed from the glove box and placed in the physical vapor deposition (PVD) system (Angstrom Engineering) for cathode sputtering through the shadow mask. An aluminum cathode (100 nm) was deposited by e‐beam sputtering with 2 Å/s at 1.5 × 10^−7^ Torr. 8 pixels with a 2 × 2 mm^2^ emitting area were formed on each substrate. Each device was encapsulated in UV‐curable epoxy resin, covered with 0.2 mm thick glass (OLEDs based on glass substrates) or 0.05 mm thick PET film (OLEDs based on PET substrates), for 5 min using the UV Curing LED System (Thorlabs).

Analysis of luminance and current density was performed with a LED measurement system (Ossila). Voltage was changed from 0 to 10 V.

### 4.7 Fabrication of Gold Electronic Tattoos

As‐synthesized Au/Cu substrates with gold thicknesses of 5 nm and 20 nm were spin coated (2500 rpm for 60 s) with a PMMA support layer (PMMA 950 A4), followed by baking at 200 °C for 20 min, yielding a PMMA thickness of ∼200 nm. To remove the Cu substrate, the PMMA/Au/Cu stack was floated on a 0.1 M ammonium persulfate (APS; Sigma‐Aldrich) aqueous solution until complete etching was achieved. The resulting freestanding PMMA/Au film was cleaned by sequentially floating on deionized (DI) water three times for 15 min each to eliminate residual etchant. The cleaned PMMA/Au film was transferred onto commercial tattoo paper (Silhouette Temporary Tattoo Paper). To orient the Au surface upward, a secondary transfer step was performed by flipping the film and placing it onto a second tattoo paper, following the method described by Kireev et al. [50]. After drying overnight, the Au/tattoo composite was patterned into rectangular electrode arrays (5  ×  8 mm; effective skin‐contact area: 5  ×  5 mm) using a precision desktop mechanical cutter (Silhouette Cameo 4 Plus), rendering them ready for epidermal electrophysiological applications.

### 4.8 Au Tattoo Transfer and Skin‐Impedance Measurement

For on‐skin testing, the patterned Au tattoos (5 × 8 mm) on their tattoo paper backing were first soaked in DI water for ∼30 min to facilitate their release. To establish electrical contact between each Au tattoo and the external readout circuitry, a 10 µm‐thin‐flexible adhesive tape (Iwatani, ISRBSMK10G) coated with Ti/Au (5 nm/100 nm) was attached to the skin at the intended electrode site. One end of this conductive tape was connected to a lead wire, while the other end served as a contact pad for the Au tattoo. After the tape was in place, the water‐soaked tattoo paper was positioned on the skin (overlapping the exposed metal pad of the tape) and gently pressed. The backing tattoo paper then carefully slid off, leaving the ultrathin Au tattoo conformally adhered to the skin. Figure 5a illustrates the on‐skin transfer process and the impedance measurement set up.

Electrode‐skin impedance measurements were performed using a Hioki IM3536 LCR meter operated in constant‐voltage mode (50 mV AC, no DC bias). Impedance spectra were recorded over a frequency range of 10 Hz to 1 MHz, and each measurement was repeated three times to ensure reproducibility, each data point was measured four times and averaged instrumentally. The reported impedance values correspond to the total impedance measured per tattoo between a pair of identical Au tattoo electrodes on the skin, at frequency of 10 kHz (Figure 5c).

### 4.9 Measurement Setup and Signal Processing

Electrophysiological recordings, comprising electrocardiography (ECG) and surface electromyography (sEMG), were collected utilizing the OpenBCI Cyton board which was reliably used for accurate ECG and HRV measurement. To acquire signals, commercially available Cardinal Health Ag/AgCl gel electrodes were trimmed to a uniform size of 5 × 8 mm to match the surface area of the gold electrodes used for comparison. All signals were exported in raw format and then were passed through an offline preprocessing chain to remove artifacts and extract important information while maintaining physiological integrity. To eliminate temporal distortion, zero‐phase filtering was used, which was accomplished by applying digital filters from forward‐backward sides of the signal window, resulting in symmetrical phase response. To reduce baseline drift and high‐frequency noise while preserving the QRS complex and other low‐frequency components, ECG signals were bandpass filtered with a 4th‐order Butterworth filter with cutoffs of 1–30 Hz. sEMG signals were filtered with a 20–120 Hz bandpass filter to capture average action potential due muscle activation in the region and remove motion artifacts. A 60 Hz notch filter was also applied to reduce power‐line interference.

Power spectral density (PSD) was calculated using the Welch method, which averaged overlapping windowed segments to produce smooth and statistically accurate frequency‐domain representations. The dominant ECG frequency content was focused between 1–2 Hz, but sEMG signals revealed broadband energy throughout 60–120 Hz during active muscular contraction. Baseline noise parameters were determined from quiescent segments with no physiological activity using time‐domain metrics such as root mean square, standard deviation and low‐frequency spectral energy analysis.

The signal‐to‐noise ratio (SNR) was calculated by comparing the amplitude of physiological signal features. R‐peak amplitude for ECG and RMS envelope during contraction for sEMG with baseline noise metrics generated from stable resting segments. Artifacts including movement‐induced peaks and saturation were eliminated using amplitude thresholding and derivative‐based spike identification. This pipeline allowed for the extraction of ECG and EMG signals using the fabricated gold electronic tattoos and the gel electrodes.

## Author Contributions

D.Y., M.M., G.E., and A.K. contributed equally to this work. D.Y., M.M., A.A., and V.V. suggested the project. A.N.G., D.K., A.A., K.S.N., and V.V. directed the project. D.Y., M.M., G.E., A.K., A.S., P.N., P.C., I.K., A.M., G.T., D.G., M.S., N.P., H.Z., V.G.K., and D.K. performed the measurements and analyzed the data. D.Y. and M.M. prepared the samples. D.Y., A.V., I.K., and N.O. provided theoretical support. D.Y. and G.E. wrote the original manuscript. D.Y., G.E., M.M., A.K., A.N.G., D.K., P.N., P.C., A.A., K.S.N., and V.V. reviewed and edited the paper. All authors contributed to the discussions and commented on the paper.

## Conflicts of Interest

The authors declare no conflicts of interest.

## Supporting information




**Supporting File**: advs76495‐sup‐0001‐SuppMat.docx.

## Data Availability

The datasets generated during and/or analysed during the current study are available from the corresponding author upon reasonable request.
