# Supplementary material for: Wafer‐Scale Atomically‐Thin Gold: Transferable Platform for Flexible Optoelectronics, Thermal Management and Bio‐Interfaces
Source: Adv Sci (Weinh). 2026 Aug 4;13(55):e76495. doi: 10.1002/advs.76495 (PMC13435944; doi:10.1002/advs.76495)
Supplement: Supplementary file 1 [file ADVS-13-e76495-s001.docx]

**Supplementary Information for**

**Wafer-scale atomically-thin gold: transferable platform for flexible optoelectronics, thermal management and bio-interfaces**

Dmitry Yakubovsky^1,#^, Mikhail Mironov^1,#^, Georgy Ermolaev^1,#^, Aleksandr Khrebtov^1,#^, Aleksandr Slavich^1^, Prashant Narute^2^, Pritom Chowdhury^2^, Roman Kirtaev^1^, Igor Khramtsov^1^, Anton Minnekhanov^1^, Gleb Tselikov^1^, Dmitriy Grudinin^1^, Maria Sidorova^1^, Nikolay Pak^1^, Pavel Maslov^1^, Andrey Vyshnevyy^1^, Hongkai Zhou^3^, Vasyl G. Kravets^3^, Alexander N. Grigorenko^3^, Ivan Kruglov^1^, Nikita Orekhov^1^, Dmitry Kireev^2,*^, Aleksey Arsenin^1^, Kostya S. Novoselov^4,5,6,*^, Valentyn Volkov^1,*^

*^1^Emerging Technologies Research Center, XPANCEO, Internet City, Emmay Tower, Dubai, United Arab Emirates*

*^2^Department of Biomedical Engineering, University of Massachusetts, Amherst, Amherst, MA, 01003, USA*

*^3^Department of Physics and Astronomy, University of Manchester, Manchester, UK*

*^4^The University of Manchester, National Graphene Institute, Oxford Rd, Manchester M13 9PL, UK*

*^5^Department of Materials Science and Engineering, National University of Singapore, Singapore 03-09 EA, Singapore*

*^6^Institute for Functional Intelligent Materials, National University of Singapore, Building S9, 4 Science Drive 2, Singapore 117544, Singapore*

*^#^These authors contributed equally*

**Correspondence should be addressed to the e-mails:* [*vsv@xpanceo.com*](mailto:vsv@xpanceo.com)*,* [*kostya@nus.edu.sg*](mailto:kostya@nus.edu.sg)*,* [*dkireev@umass.edu*](mailto:dkireev@umass.edu)

**Table of Сontents**

**Supplementary Note 1: Molecular dynamics modeling of Au deposition**

**Supplementary Note 2: Morphology characterization of ultrathin gold films and copper foil**

**Supplementary Note 3: Flexible organic light-emitting diodes (OLEDs) fabrication**

**Supplementary Note 4: Ultrathin gold electronic tattoos fabrication**

**Supplementary Note 1: Molecular dynamics modeling of Au deposition**

**Preparation of MD potential**

MD simulations were performed with MTP potential [1] version 2. Forces and energies for atomic configurations from the dataset were obtained using VASP package [2] by employing DFT with Perdew-Burke-Ernzerhof (PBE) exchange-correlation functional [3], a 350 eV energy cutoff for electrons and a 3x3x3 *k*-point mesh. Atomistic configurations used in datasets (dataset_AuSi.cfg and dataset_AuCu.cfg), file containing trained MTP potential (AuSi.mtp and AuCu.mtp) as well as python scripts used to process the output from Quantum Espresso are available at our github page [4]. The resulting accuracy for energy reached 2.9 meV/atom (1.7 meV/atom on train dataset) and 10.8 meV/atom (5.4 mev/atom on train dataset) for Au-Cu and Au-Si systems respectively (Supplementary Figure 1).

**Supplementary Figure 1. a,** Energy and **b,** force parity plots for the Au-Cu system.

**Modeling of Au deposition on Si substrate**

All MD calculations were performed using the Large-scale Atomic/Molecular Massively Parallel Simulator (LAMMPS) program package [5] with machine-learning MTP interatomic potential.

While the experimental process of Au deposition on substrate takes hours, in classical MD we are limited by the timescale of hundreds of nanoseconds. To effectively model material relaxation and atom diffusion on a macroscopic timescale, we alternated molecular dynamics (MD) simulations with Monte Carlo (MC) methods (Supplementary Figure 2a). Classical MD simulations for initial Au deposition were coupled with time-stamped force-bias Monte Carlo (tfMC) for relaxation [6]. The tfMC parameters were set as follows: a maximum displacement length of 0.2 Å and an effective temperature of *T* = 300 K.

During the MD stage Au atoms were created in a gas phase and launched towards the substrate with a kinetic energy corresponding to 300 K. The substrate itself was divided into three section: atoms in the bottom part were frozen, atoms in the middle were kept at constant *T* = 300 K temperature via Nose-Hoover thermostat [7] and atoms on the top were kept in micro canonical ensemble (NVE) (Supplementary Fig. 2b). The surface area of the substrate was 5.7 x 5.7 nm^2^ and periodic boundary conditions were applied along X and Y axes. The system was visualized using the OVITO package [8].

**Supplementary Figure 2. Molecular dynamics simulation of non-isotropic Au deposition.** **a,** Protocol of Au atoms deposition with subsequent steps of MD relaxation at finite temperature (NVE regime) and Monte-Carlo stage. **b,** Computational cell containing Cu substrate and deposited Au atoms. Gold deposited on **c,** Cu, **d,** Si substrate. **e,** Scheme for structure factor calculation. **f,** Structure factor for gold from bottom to top layers.

**Gold film crystallinity on different substrates**

We analysed the level of crystallinity of Au film after the growth of 6 atomic layers on Cu and Si substrates (Supplementary Figure 2c-d). The deposition scheme was identical to one described in “Modeling of Au deposition” section. Structure factor $\eta$ for atom *i* belonging to a particular layer was calculated as followed: $\eta=\left\langle\sin\left( k\left( \theta\right)r \right) \right\rangle^{2}+\left\langle\cos\left( k\left( \theta\right)r \right) \right\rangle^{2}$, where *k* – is the minimal vector of a reciprocal lattice laying in the plane of the layer (Supplementary Figure 2e). For ideal lattices $\eta$ is equal to 1, while for disordered structures it tends to zero.

The results show that on Cu substrate gold reaches its maximum $\eta$ ≈ 0.9-0.93 already within the first layer (Supplementary Figure 2f). It means that structurally even films consisting of one or two atomic layers preserve high crystallinity. For Si substrate, on the other hand, we much more defective structure with $\eta$ ≈ 0.3-0.5 for the first two atomic layers.

**Supplementary Note 2: Morphology characterization of ultrathin gold films and copper foil**

To clearly illustrate the different growth outcomes, we visualised the surface morphology of ultrathin gold films of different thicknesses (from 2 to 8 nm) grown on copper foil, an exfoliated MoS_2_ crystal, and a SiO_2_/Si substrate. As seen in Supplementary Figure 3, high-resolution scanning electron microscopy (HR-SEM) images clearly demonstrate three distinct morphologies of ultrathin gold: island-like (Au-SiO_2_), percolated (Au-MoS_2_) and continuous (Au-Cu).

**Supplementary Figure 3.** HR-SEM surface morphology images of gold ultrathin films for thicknesses from 2 to 8 nm grown on different substrate materials (all scale bars are 100 nm). In “Au-Cu” gold films were grown on anatomically-smooth copper foil and transferred onto SiO_2_/Si substrate.

Atomic force microscopy (AFM) characterization confirmed an atomically-smooth surface of the freshly template-stripped copper foil, with a root-mean-square (RMS) surface roughness not exceeding 0.45 nm over AFM scans 10×10 μm^2^ area and 0.16 nm over 1×1 µm^2^, which are presented in Supplementary Figure 4. As a result, AFM revealed micron-scale domains consistent with high surface flatness and crystallinity.

**Supplementary Figure 4.** Surface morphology characterization of template stripped copper foil by AFM surface imaging of areas: 10×10 μm^2^, 4×4 μm^2^ and 1×1 μm^2^ with RMS roughness determination across the area.

AFM analysis of the copper foil surface (area of 0.5×0.5 µm^2^) after 1 nm-nominal-thick gold deposition on Cu did not reveal the formation of visible islands: RMS roughness remained virtually unchanged compared to the freshly template-stripped Cu foil (RMS = 0.16 nm), confirming continuous 2D gold growth (see Supplementary Figure 5a). In contrast, for 1 nm-thick gold deposited on SiO_2_ and MoS_2_ AFM revealed a several-fold increase in RMS due to island formation consistent with 3D Volmer-Weber growth, presented in Supplementary Figure 5b-c.

**Supplementary Figure 5.** AFM scans at a scale of 0.5×0.5 μm^2^ demonstrating the roughness of (a) Au on SiO_2_ and (d) Au on MoS_2_ after 1 nm-thick gold deposition (all scale bars are 100 nm).

Thicknesses of gold films transferred onto SiO_2_/Si were verified by AFM direct step-height measurements. For the nominal thicknesses of the transferred gold 2 nm and 8 nm an AFM profile in a crack region yields approximately 2.5 nm and 8.2 nm (see Supplementary Figure 6), due to the influence of roughness at the bottom interface.

**Supplementary Figure 6. a,** Thickness measurement of 8 nm-thick gold film, transferred on SiO_2_/Si, by direct AFM scan of the scratched area with **b,** the line thickness profile and approximation result of step-height (8.2 ± 0.4 nm), which confirms the values of mass-equivalent thickness.

Fabricating an ultrasmooth copper substrate for gold growth using the template stripping method is necessary to minimize defects (cracks, voids, and wrinkles) in transferred film and to achieve a more uniform, continuous ultrathin gold film. Because the defect density becomes critical at thicknesses of only a few nanometers, reducing the morphological defect density allows the transfer of continuous gold films at smaller thicknesses (down to 2 nm). In addition to achieving atomically smooth (RMS < 0.3 nm) surface roughness of the copper, we found that the improved quality of the resulting film is primarily associated with a more crystalline substrate structure, specifically, a reduction in grain boundaries on the copper surface, as can be seen from the comparison in Supplementary Figure 7. Here, the influence of the copper surface morphology used for gold growth and transfer of 8 nm-thick gold film on its uniformity and conductivity is demonstrated. This comparative figure clearly illustrates the advantage of the using template stripping method.

In addition, Supplementary Figure 8 includes commercial rolled copper foil, whose RMS roughness exceeds that of the template-stripped foil by more than an order of magnitude even over a small 2×2 µm^2^ window, confirming that commercial stock cannot serve as a growth template for continuous gold at the few-nanometre thicknesses reached here. A secondary benefit of the template-stripping route is that the gold-facing copper surface remains shielded by the silicon wafer until loading into the evaporator, which limits the native oxide that would otherwise be trapped beneath the gold and promotes local island growth.

**Supplementary Figure 7.** Surface morphology characterization of the initial copper substrate and gold 8 nm-thick ultrathin films after the graphene-inspired transfer process. (a–b) SEM and AFM surface images of a 500 nm-thick Cu layer deposited onto a Si substrate at room temperature (a) and at 250°C (b), along with the morphology of the Au film transferred by the method used in [9] [Nano Lett., 2024, 10.1021] from the corresponding substrate; the obtained RMS roughness values are indicated. (c) SEM and AFM surface images of template-stripped Cu foil and the resulting Au ultrathin film, with the obtained sheet resistance of the Au films and RMS roughness values. Scale bars in all SEM images are 100 nm, and in AFM images are 500 nm.

**Supplementary Figure 8**. Comparison of copper foil and transferred gold films surface morphologies. (a) AFM image of commercial rolled Cu foil ("graphene CVD-grade"): RMS = 4.5 nm over a 2.0×2.0 µm^2^, and (b) AFM surface image of the template-stripped Cu foil produced in this work: RMS = 0.2 nm over a 2.0×2.0 µm^2^ area. (c–d) The resulting Au ultrathin films with thickness of (c) 5.5 nm (Mironov et al., Nano Lett. 24, 16270, 2024) and (d) 5 nm (from the new paper) with the measured RMS roughness and sheet resistance, transferred from correspondent Cu substrates.

**Supplementary Note 3: Flexible organic light-emitting diodes (OLEDs) fabrication**

**Materials**

ITO glass substrates (20 Ω/sq, thickness 1.1 mm), poly(9,9-di-n-octylfluorenyl-2,7-diyl) PFO (Mw = 260 817), poly(3,4-ethylenedioxythiophene) polystyrene sulfonate PEDOT PSS (Clevios P VP AI 4083), 8-hydroxyquinolinolato-lithium Liq were purchased from Ossila. Poly(9,9-dioctylfluorene-alt-benzothiadiazole) F8BT (Mw > 20 000) was purchased from Lumtec. PFO and F8BT were dissolved in toluene (PFO:F8BT = 95:5, overall concentration 10 mg/ml). Liq was dissolved in isopropyl alcohol (1 mg/ml). All solutions were previously filtered by 0.45 μm PTFE syringe filters.

**Device fabrication and characterization**

ITO glass substrates (20 Ω/sq, thickness of 1.1 mm, Ossila) were subsequently cleaned in the ultrasonic bath with acetone (5 min) and isopropanol (5 min). ITO substrates and substrates coated with gold films were treated in UV Ozone cleaner (Ossila) for 30 min prior to the coating of the PEDOT:PSS layer and placed in the glove box (Ossila) with N_2_ atmosphere. All substrates were subsequently coated with PEDOT:PSS, PFO:F8BT and Liq layers by spin coater (Ossila) and baked on a hot plate at 90 °C for 10 min after each coating step. After that, substrates were removed from the glove box and placed in the physical vapor deposition (PVD) system (Angstrom Engineering) for cathode sputtering through the shadow mask. Aluminum cathode (100 nm) was deposited by e-beam sputtering with 2 Å/s at 1.5×10^-7^ Torr. 8 pixels with 4×4 mm2 emitting area were formed on each substrate. Each device was encapsulated by UV-curable epoxy resin covered by 0.2 mm thick glass (OLEDs based on glass substrates) or 0.05 mm thick PET film (OLEDs based on PET substrates) for 5 min using the UV Curing LED System (Thorlabs). The device structure is shown in Supplementary Figure 7.

Analysis of luminance and current density were performed with a LED measurement system (Ossila). Voltage was changed from 0 to 10 V.

**Supplementary Figure 7. a,** Device structure of solution-processed OLEDs based on gold film and ITO anodes. **b,** Energy band diagram of the devices. The work function of the ultrathin Au anode is approximately -5.1 eV, whereas that of the conventional ITO anode is -4.9 eV. Both values align favorably with the Highest Occupied Molecular Orbital (HOMO) level of the PEDOT:PSS hole-injection layer (-5.2 eV).

**Supplementary Note 4: Ultrathin gold electronic tattoos fabrication**

**Fabrication process of ultrathin Au tattoos**

Supplementary Figure 8 shows sequential photographic images illustrating the key steps involved in the fabrication of ultrathin Au tattoos. The as-fabricated Au/Cu samples (Supplementary Figure 8a) were already coated with a 90–100 nm thick PMMA support layer. However, this initial layer was insufficient to prevent cracking during the transfer process due to its limited thickness. To ensure the mechanical stability during transfer, an additional PMMA layer (PMMA-950 A4) was spin-coated onto the samples at 2500 rpm for 60 s, followed by curing at 200 °C for 20 min, resulting in a final PMMA thickness of approximately 200 nm. The total thickness (already coated + additional coating) would be ~300 nm. As-fabricated samples were sized 2.5 x 2.5 ± 0.5 cm, with different gold thicknesses of 5 and 20 nm for comparison. For tattoo fabrication, the additional PMMA-coated ultrathin Au samples (Supplementary Figure 8b) were floated on an ammonium persulfate (APS, Sigma-Aldrich) aqueous solution (0.1 M; 22.8 g/L) to etch away the copper substrate. After complete etching, the PMMA/Au stack was rinsed three times (15 minutes each) by floating on deionized water to eliminate residual etchant. Subsequently, the cleaned PMMA/Au film was transferred onto commercial tattoo paper (Silhouette Temporary Tattoo Paper). To orient the Au surface upward, a secondary transfer step was performed by flipping the film and placing it onto a second tattoo paper, following the method described by Kireev et al. [9]. After drying (overnight), the resulting Au/tattoo structures were patterned into rectangular electrodes (5 x 8 mm; effective skin-contact area = 5 x 5 mm) using a precision desktop mechanical cutter (Silhouette Cameo 4 Plus), preparing them for electrophysiological measurements.

**Supplementary Figure 8. Step-by-step fabrication process of ultrathin Au tattoos. a,** As-fabricated Au/Cu foil with a PMMA support layer. **b,** After spin-coating and baking an additional PMMA layer for improved structural integrity. **c,** Initiation of Cu etching using 0.1 M ammonium persulfate (APS) solution. **d,** Complete Cu etching, leaving the floating PMMA/Au film on APS solution. **e,** Rinsing process to remove APS residues by floating on deionized water. **f,** Transfer of PMMA/Au stack onto tattoo paper. **g,** Second transfer to ensure Au is oriented upwards, followed by drying. **h–j,** Patterning the dried Au tattoo into desired rectangular shapes using a tabletop mechanical cutting plotter.

**Ultrathin Au tattoo transfer and skin-impedance measurement**

For on-skin testing, pre-patterned ultrathin Au tattoos (5 × 8 mm) were soaked in deionized (DI) water for approximately 30 minutes prior to transfer to facilitate their release. To establish an electrical connection between the Au tattoos and the readout circuitry (Hioki IM3536 LCR meter), a thin (~10 μm) adhesive tape (Iwatani, ISRBSMK10G) coated with Ti/Au (5/100 nm) was attached to the skin at the intended electrode site. This conductive tape was attached to the skin where one end is connected to a lead wire, while the other end served as a contact pad for the Au tattoo interface. Following attachment of the conductive tape, the water-soaked tattoo paper was brought into contact with the skin (overlapping the exposed metal pad of the tape), gently pressed, and the backing tattoo paper was slid away, leaving the Au tattoo adhered to the skin. Supplementary Figure 9 shows results of on-skin transfer and impedance measurement setup.

Electrode-skin impedance was measured using a Hioki IM3536 LCR meter operated in constant voltage mode with an AC amplitude of 50 mV and no DC bias. The experiment was conducted with the frequency sweep ranging from 10 Hz to 1 MHz, and each measurement was repeated three times to ensure reproducibility, each data point was measured four times and averaged instrumentally. The reported impedance values correspond to the total impedance between a pair of identical Au tattoo electrodes on the skin (see Supplementary Figure 9). To calculate the impedance per individual tattoo, the measured value at a representative frequency (typically 10 kHz) was divided by two, assuming symmetrical electrode-skin contact characteristics.

**Supplementary Figure 9.** Au tattoo electrodes on-skin transfer and impedance measurement setup.

**Electrophysiology monitoring measurement setup and signal processing**

Electrophysiological recordings, comprising electrocardiography (ECG) and surface electromyography (sEMG), were collected utilizing the OpenBCI Cyton board which was reliably used for accurate ECG and HRV measurement [10]. It is a wireless biopotential acquisition system equipped with a 24-bit analog front-end designed for low-noise differential signal acquisition [11].

To acquire signals, commercially available Cardinal Health Ag/AgCl gel electrodes were trimmed to a uniform size of 5 mm × 8 mm to match the surface area of the gold electrodes used for comparison. This standardization was necessary to ensure that variations in electrode-skin contact impedance were due to material qualities rather than geometrical discrepancies. Because electrode area influences contact impedance and capacitive coupling, keeping equal surface areas allowed for the isolation of material-dependent impedance and compared them side-by-side. Gel electrodes showed typical impedance of ~5–7 kΩ at a frequency of 10 kHz. To achieve similar skin conditions, the Au and Gel electrodes were put adjacent to one another on the same site. To reduce motion-induced artifacts during ECG data acquisition, electrodes were placed in a bipolar pattern on both wrists, with a reference electrode on the elbow. For EMG acquisition, electrode pairs were placed longitudinally throughout the forearm with a 3 cm inter-electrode spacing, with the reference electrode remaining on the elbow.

All signals were exported in raw format and then were passed through an offline preprocessing chain to remove artifacts and extract important information while maintaining physiological integrity. To eliminate temporal distortion, zero-phase filtering was used, which was accomplished by applying digital filters from forward-backward sides of the signal window, resulting in symmetrical phase response. To reduce baseline drift and high-frequency noise while preserving the QRS complex and other low-frequency components, ECG signals were bandpass filtered with a 4th-order Butterworth filter with cutoffs of 1-30 Hz. sEMG signals were filtered with a 20-120 Hz bandpass filter to capture average action potential due muscle activation in the region and remove motion artifacts. A 60 Hz notch filter was also applied to reduce power-line interference.

Power spectral density (PSD) was calculated using the Welch method, which averaged overlapping windowed segments to produce smooth and statistically accurate frequency-domain representations. The dominant ECG frequency content was focused between 1-2 Hz, but sEMG signals revealed broadband energy throughout 60-120 Hz during active muscular contraction. Baseline noise parameters were determined from quiescent segments with no physiological activity using time-domain metrics such as root mean square, standard deviation and low-frequency spectral energy analysis. The signal-to-noise ratio (SNR) was calculated by comparing the amplitude of physiological signal features. R-peak amplitude for ECG and RMS envelope during contraction for sEMG with baseline noise metrics generated from stable resting segments. Artifacts including movement-induced peaks and saturation were eliminated using amplitude thresholding and derivative-based spike identification. This pipeline allowed for the extraction of ECG and EMG signals using the fabricated gold electronic tattoos and the gel electrodes.

**References**

[1] Shapeev, V. Moment tensor potentials: A class of systematically improvable interatomic potentials, *Multiscale Modeling & Simulation* **14** (3), 1153–1173 (2016).

[2] Kresse, G., Joubert, D. From ultrasoft pseudopotentials to the projector augmented-wave method. *Phys. Rev. B* **59**, 1758 (1999).

[3] Perdew, J. P., Burke, K., Ernzerhof, M. Generalized gradient approximation made simple, *Phys. Rev. Lett*. **77** (18), 3865 (1996).

[4] https://github.com/interatomic-mipt/Au_Si_deposition/

[5] Thompson, A. P., Aktulga, H. M., *et al.* Lammps-a flexible simulation tool for particle-based materials modeling at the atomic, meso, and continuum scales. *Computer Physics Communications* **271**, 108171 (2022).

[6] Neyts, E. C., Bogaerts, A. Combining molecular dynamics with Monte Carlo simulations: implementations and applications. *Theor. Chem. Acc.* **132**, 1320 (2013).

[7] Nose, S. A unified formulation of the constant temperature molecular dynamics methods. *J. Chem. Phys.* **81**, 511–519 (1984).

[8] Stukowski, A. Visualization and analysis of atomistic simulation data with ovito–the open visualization tool. *Modelling Simul. Mater. Sci. Eng.* **18**, 015012 (2010).

[9] Kireev, D., Ameri, S.K., Nederveld, A. *et al.* Fabrication, characterization and applications of graphene electronic tattoos. *Nat. Protoc.* **16**, 2395–2417 (2021).

[10] Burma, J. S. *et al.* The validity and reliability of an open source biosensing board to quantify heart rate variability. *Heliyon* **7**, 6, e07148 (2021).

[11] Cardona-Álvarez, Y. N., Álvarez-Meza, A. M. *et al.* A Novel OpenBCI Framework for EEG-Based Neurophysiological Experiments. *Sensors* **23** (7), 3763 (2023).
